# New insights into the role of dipeptidyl peptidase 8 and dipeptidyl peptidase 9 and their inhibitors

**DOI:** 10.3389/fphar.2022.1002871

**Published:** 2022-09-12

**Authors:** Chenkai Cui, Xuefei Tian, Linting Wei, Yinhong Wang, Kexin Wang, Rongguo Fu

**Affiliations:** ^1^ Department of Nephrology, Second Affiliated Hospital of Xi’an Jiaotong University, Xi’an, China; ^2^ Section of Nephrology, Department of Internal Medicine, Yale University School of Medicine, New Haven, CT, United States

**Keywords:** DPP8, DPP9, cell behavior, inflammation, pyroptosis, cancer, organ fibrosis, immune regulation

## Abstract

Dipeptidyl peptidase 8 (DPP8) and 9 (DPP9) are widely expressed in mammals including humans, mainly locate in the cytoplasm. The DPP8 and DPP9 (DPP8/9) belong to serine proteolytic enzymes, they can recognize and cleave N-terminal dipeptides of specific substrates if proline is at the penultimate position. Because the localization of DPP8/9 is different from that of DPP4 and the substrates for DPP8/9 are not yet completely clear, their physiological and pathological roles are still being further explored. In this article, we will review the recent research advances focusing on the expression, regulation, and functions of DPP8/9 in physiology and pathology status. Emerging research results have shown that DPP8/9 is involved in various biological processes such as cell behavior, energy metabolism, and immune regulation, which plays an essential role in maintaining normal development and physiological functions of the body. DPP8/9 is also involved in pathological processes such as tumorigenesis, inflammation, and organ fibrosis. In recent years, related research on immune cell pyroptosis has made DPP8/9 a new potential target for the treatment of hematological diseases. In addition, DPP8/9 inhibitors also have great potential in the treatment of tumors and chronic kidney disease.

## 1 Introduction

Dipeptidyl peptidases (DPPs) belong to the S9b serine protease family with a conserved catalytic triad of serine, aspartate, and histidine (Olsen and Wagtmann, 2002), which can cleave the N-terminal dipeptide of their substrate if proline is at the penultimate position. The key members of the DPP family include DPP4, DPP8, DPP9, and FAP. DPP4 predominantly exists in soluble and membrane-bound forms in the body. Since DPP4 inhibition can protect incretin hormones from degradation, DPP4 inhibitors have been widely used in the treatment of type 2 diabetes (Baggio and Drucker, 2007), and studies have also found that DPP4 plays important roles in cell death regulation, immunity, metabolic regulation, tumorigenesis, and other processes (Duan et al., 2017; Enz et al., 2019). The research on FAP mainly focuses on cancer (Pure and Blomberg, 2018). In recent years, DPP8 and DPP9 have attracted increasingly extensive attention since DPP8/9 inhibitors have been found to induce immune cell pyroptosis (Okondo et al., 2017). DPP8 and DPP9 have very high similarities in both sequence and structure. They are widely expressed in animals with major localization in the cytoplasm and nucleus, and sometimes in the plasma membrane. However, their functions remain to be determined. DPP8/9 may play a role through its cleavage of substrates. Many of the DPP8/9 substrates currently have been shown to be related to immunity and energy metabolism, such as some cytokines and adenylate kinase 2 (AK2) (Ajami et al., 2008; Zhang et al., 2015a; Finger et al., 2020). DPP8/9 can also function by interacting with proteins such as nucleotide-binding domain and leucine-rich repeat pyrin-domain containing protein 1 (NLRP1) (Huang et al., 2021a; Hollingsworth et al., 2021) and caspase activation and caspase recruitment domain-containing protein 8 (CARD8) (Sharif et al., 2021). Burgeoning evidence have shown that DPP8/9 plays essential roles in various biological processes and diseases, such as tumor development, immune regulation, inflammatory response, energy metabolism, cell proliferation and apoptosis, and cell adhesion and migration. Recent research findings from us have shown that DPP8/9 exerts an important role in renal tubulointerstitial fibrosis *in vivo* and *in vitro*, which could be markedly mitigated by DPP8/9 inhibitors (Zhang et al., 2021).

In this review, we summarize the molecular characteristics, distribution, functions, and implications of DPP8/9 on relevant diseases. We highlight the new insights of DPP8/9 in pyroptosis, cancer, and organ fibrosis, as well as the research progress of selective DPP8/9 inhibitors. This review aims to gain a deeper understanding of DPP8/9 and possible application of their inhibitors for relevant diseases treatment.

## 2 The distribution and substrates of DPP8/9

### 2.1 Molecular expression, distribution, and cellular localization

The DPPs family is mainly constituted of DPP4, DPP8, DPP9, and FAP. DPP8 and DPP9 are ubiquitously expressed in mammals including humans and are expressed in the immune system, epithelium, endothelium, nervous system, reproductive organs, and various other cells (Olsen and Wagtmann, 2002; Qi et al., 2003; Ajami et al., 2004; Maes et al., 2007; Dubois et al., 2009; Tang et al., 2009; Yu et al., 2009; Matheeussen et al., 2011; Harstad et al., 2013; Matheeussen et al., 2013). DPP9 is the only DPPs family enzyme detectable in human carotid endothelial cells (Matheeussen et al., 2011). Some studies have also found that DPP9 is localized to the bronchi (Schade et al., 2008; Baginski et al., 2011) and skin (Yu et al., 2009; Harstad et al., 2013; Gabrilovac et al., 2017). In addition, DPP8/9 was found to be localized in spermatozoa in bovine and rat testis (Dubois et al., 2009), and a natural short form of DPP9 was purified from bovine testis (Dubois et al., 2010).

The genes of *DPP8* and *DPP9* are located on human chromosomes 15q22 and 19p13, respectively. Through cloning, sequencing, and silico identification analysis of these two proteases, it was found that they have considerable sequence and structural homology with DPP4. DPP8/9 also exists in the form of dimers, containing β-propeller and α/β hydrolase domains, showing similar activity to that of DPP4, with the ability to cleave prolyl bonds (Abbott et al., 2000; Olsen and Wagtmann, 2002; Ajami et al., 2004). However, the findings from the further crystal and molecular structure studies indicated that DPP8/9 and DPP4 have significant differences in the structure of the ligand-binding site (Ross et al., 2018). At the same time, both DPP8 and DPP9 have very high sequence similarities to each other (77% amino acid similarity, 57% amino acid identity, and higher similarity within the active site) (Van Goethem et al., 2011). Notably, some studies have found that the cleavage sites, localization, and physiological and pathological processes of the two involved are not identical. For example, the dipeptide Val-Ala is a common cleavage site for DPP9, but DPP8 cannot recognize it (Zhang et al., 2015a).

**FIGURE 1:**
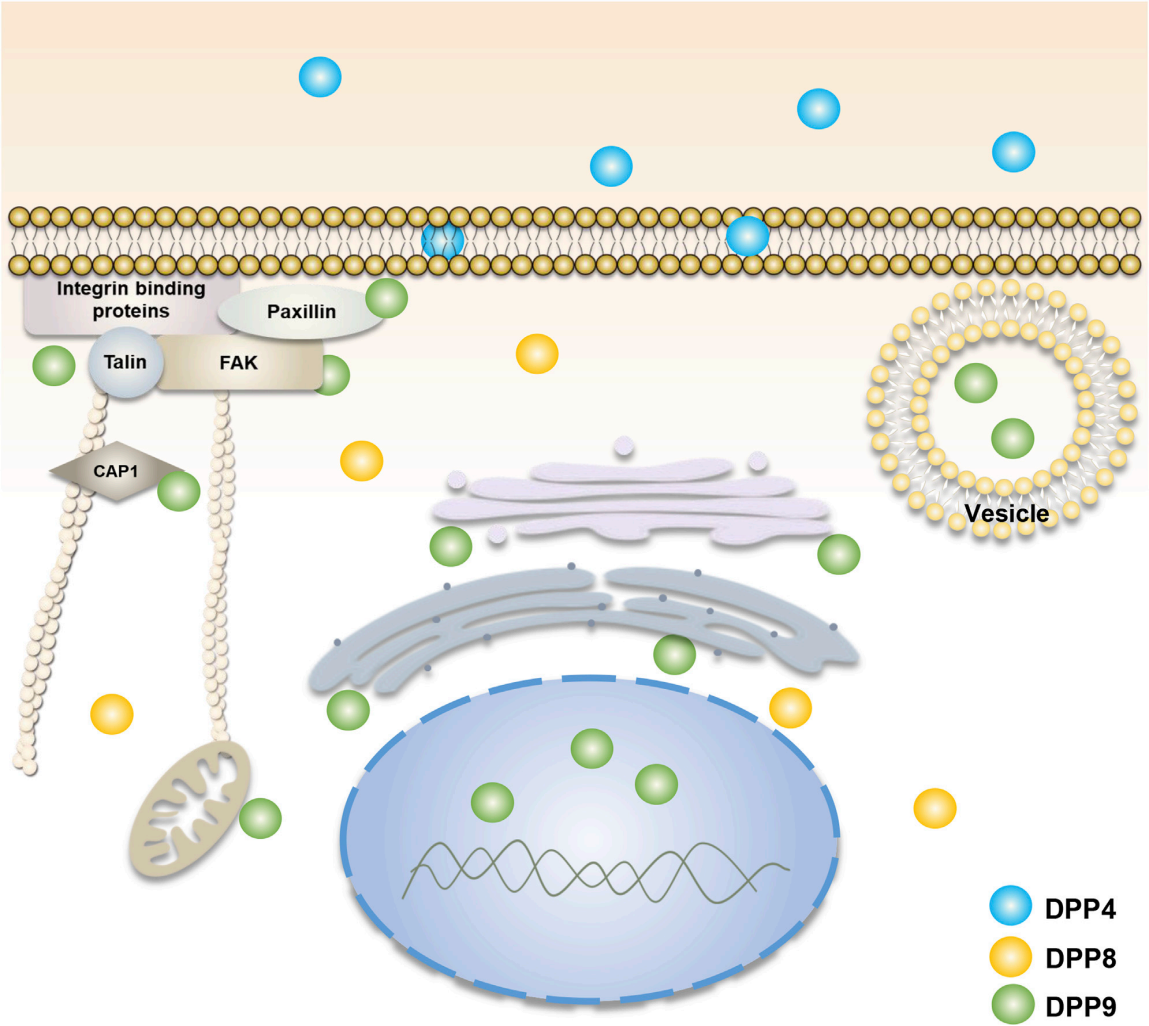
Cellular localization of DPP4, DPP8, and DPP9. DPP4 (blue) is mainly found in cell membranes and body fluids. DPP8 (yellow) and DPP9 (green) are mainly present in the cytoplasm (Olsen and Wagtmann, 2002; Abbott et al., 2000). The function of DPP9 is involved in cell adhesion and migration (Zhang et al., 2015b; Gabrilovac et al., 2017), energy metabolism (Finger et al., 2020; Wilson et al., 2013), and the processing of antigenic peptides (Geiss-Friedlander et al., 2009; Zapletal et al., 2017). Its localization is associated with focal adhesions, endoplasmic reticulum, Golgi apparatus, mitochondria and vesicles. The long-form DPP9 is localized to the nucleus (Justa-Schuch et al., 2014). DPP8/9 can be present in the cell membrane in DPP4-deficient immune cells (Bank et al., 2011; Buljevic et al., 2018).

Unlike DPP4, DPP8 and DPP9 lack transmembrane regions and secretory signals and are mainly localized in the cytoplasm (Abbott et al., 2000; Olsen and Wagtmann, 2002) (Figure 1). However, some studies have revealed that DPP8 and DPP9 can exist on the surface of immune cells in the absence of DPP4 expression (Bank et al., 2011; Buljevic et al., 2018). Soluble DPP8 and DPP9 have also been found in mouse serum (Buljevic et al., 2018), suggesting that they could somehow be secreted outside the cell and function by their enzyme activity and/or by interactions with extracellular molecules. In addition, DPP9 in macrophage cells may be involved in antigen presentation and temporarily located on the plasma membrane surface (Zapletal et al., 2017). In tumor cells and fibroblasts, DPP9 may participate in cell adhesion and migration and locates adjacent to the edge of the plasma membrane (Zhang et al., 2015b; Gabrilovac et al., 2017). It has been found that there are two homologous translation products for DPP9, in which the long-form DPP9 has a long N-terminal sequence with nuclear localization signal and is preferentially located in the nucleus (Justa-Schuch et al., 2014), as such, the two homologous types of DPP9 could play different roles. In addition, some studies have also displayed that the distribution of different transcripts and expression products of DPP9 is also different *in vivo*. Among them, the 4.4 kb *DPP9* gene transcript mRNA is dominant and ubiquitously expressed, while the 5 kb transcript mRNA is mainly present in skeletal muscle (Ajami et al., 2004).

### 2.2 Regulation of DPP8/9 activity

The activity of DPP8/9 is affected by small ubiquitin-like modifier (SUMO) modification and its redox (Park et al., 2008). DPP8/9 is allosterically activated by interacting with the E67-interacting loop (EIL) in SUMO1 through a surface defined as the SUMO-binding arm (SUBA). Mutations in E67 result in loss of binding. Downregulation of SUMO1 results in a decrease in intracellular DPP9 enzymatic activity. It was also found that SUMO1-EIL peptide (SLRFLFEGQRIADNH) can dissociate the DPP9-SUMO1 complex (Pilla et al., 2012). Since then, the SUMO1-EIL peptide has also been proved to be a noncompetitive inhibitor of DPP8/9. Variants with higher inhibition efficiency can be obtained by modifying and truncating EIL peptides, such as SLRFLYEG et al. (Pilla et al., 2013), and this inhibitory effect has also been verified in the study of the crystal structure and molecular dynamics of DPP8/9 (Ross et al., 2018). In addition, the activity of DPP9 purified from bovine testis may depend on their cysteine redox state, with the activity of the enzyme being reversibly decreased when the enzyme is oxidized and increased when it is reduced (Dubois et al., 2010). Human DPP8/9 enzymatic activity is also reversibly reduced after oxidation (Park et al., 2008). However, the activity of human DPP9 was not affected by reducing agents (Tang et al., 2009).

### 2.3 Roles of DPP8/9 substrates

DPP8/9 exerts their biological functions by interacting with their specific substrates (Table 1), so identifying their substrates are very important to explore the potential roles of DPP8/9 in health and disease status. In the early stage, it was found that *in vitro* recombinant DPP8 and DPP9 can cleave some DPP4 substrates, such as glucagon-like peptide-1 (GLP1), glucagon-like peptide-2 (GLP2), neuropeptide Y (NPY), and peptide YY (PYY), with lower efficiency than DPP4. Since DPP8/9 is located intracellularly and these substrates are mainly located extracellularly, they may not be substrates for DPP8/9 to function *in vivo* (Bjelke et al., 2006; Ajami et al., 2008). However, it has been shown that NPY can be cleaved in the presence of the DPP4 inhibitor in rat brain extracts, suggesting that DPP8/9 may also participate in the cleavage of NPY *in vivo* (Frerker et al., 2007). Endogenous NPY-driven cell death in Ewing sarcoma family tumors (ESFT) can be blocked by DPPs, including DPP8/9, which also indicates that NPY can serve as an *in vivo* substrate of DPP8/9 (Lu et al., 2011).

**TABLE 1:** Roles of DPP8/9 substrates.

Enzyme	Substrate	Role	References
DPP8	IP10 (CXCL10)	Immunity	Ajami et al. (2008)
	ITAC (CXCL11)	Immunity	
	SDF-1 (CXCL12)	Immunity	
DPP9	RU1_34-42_ peptide (VPYGSFKHV)	Immunity	Geiss-Friedlander et al. (2009)
	CO7A1	Immunity	Zhang et al. (2015a)
	CXCL10	Immunity	
	IL-1RA		
	MYO1A	Protein secretory process	
	NUCB1	Immunity	
	S100-A10	Immunity	
	SET	Immunity	
	AMRP1		
	CSN8		
	Syk	Immunity	Justa-Schuch et al. (2016)
	BRCA2	DNA repair	Bolgi et al. (2022)
DPP8/9	GLP-1	Metabolism	Bjelke et al. (2006)
	GLP-2	Metabolism	
	NPY	Metabolism	
	PYY	Metabolism	
	Acetyl-CoA acetyltransferase, mitochondrial	Metabolism	Wilson et al. (2013), Finger et al. (2020)
	Adenylate kinase 2	Metabolism	
	Bifunctional purine biosynthesis protein PURH	Metabolism	
	Calreticulin	Immunity	
	Cathepsin Z/X	Proteolysis	
	Collagen-binding protein 2 (Serpin H-1)	Protein processing	
	C-1-tetrahydrofolate synthase, cytoplasmic	Metabolism	
	Dihydropyrimidine dehydrogenase [NADP+]	Metabolism	
	Endoplasmin	Immunity	
	Enoyl-CoA hydratase, mitochondrial	Metabolism	
	Heat shock 70 kDa protein 1L	Immunity	
	Mitochondrial import receptor subunit TOM34	Protein transport	
	Obg-like ATPase 1	Metabolism	
	serine/threonine-protein phosphatase 6	Cell cycle	

Abbreviation: IP10, inflammatory protein-10; ITAC, interfering T-cell chemokines; SDF-1, chemokines stromal cell-derived factor; RU1, Renal Ubiquitous Protein 1; CO7A1, Collagen alpha-1 (VII) chain; CXCL10, C-X-C motif chemokine 10; IL-1RA, Interleukin-1 receptor antagonist protein; MYO1A, Unconventional myosin-Ia; NUCB1, Nucleobindin-1; S100-A10, Protein S100-A10; SET, phosphatase 2A inhibitor I2PP2A; AMRP1, Alpha-2macroglobulin receptorassociated protein; CSN8, COP9 signalosome complex subunit 8; BRCA2, breast cancer associated protein 2; GLP1, glucagon-like peptide-1; GLP2, glucagon-like peptide-2; NPY, neuropeptide Y; PYY, peptide YY.

Other natural substrates have also been proved to be the substrates of DPP8, such as inflammatory protein-10 (IP10, CXCL10), interfering T-cell chemokines (ITAC, CXCL11), and chemokines stromal cell-derived factor (SDF-1, CXCL12) (Ajami et al., 2008). The first natural substrate of DPP9 identified was the RU1_34-42_ peptide (VPYGSFKHV), and the silencing of DPP9 in the human renal carcinoma cell line BB64-RCC resulted in the increased presentation of the RU1_34-42_ peptide to cytolytic T lymphocytes (CTLs), suggesting that DPP9 may play an important role in antigen presentation (Geiss-Friedlander et al., 2009). Novel substrates were also identified by using a 2D differential in-gel electrophoresis approach in mouse embryonic fibroblasts lacking endogenous DPP9 activity, including cytokines such as C-X-C motif chemokine 10 (CXCL10), S100-A10, SET, NUCB1, and Interleukin-1 receptor antagonist protein (IL-1RA), indicating that DPP9 may function in immune regulation and inflammatory disease (Zhang et al., 2015a). In addition, Syk is a tyrosine kinase in B cell signal transduction, which can regulate cell metabolism, migration, proliferation, apoptosis, and gene transcription. Meanwhile, DPP9 has been indicated as a negative regulator of Syk by generating a new N-terminus of Syk. The interaction between Syk and DPP9 requires Filamin A (FLNA) linkage (Justa-Schuch et al., 2016). Therefore, DPP8/9 is closely related to the regulation of immunity. By utilizing terminal amine isotopic labeling of substrates (TAILS), some substrates of DPP8/9 related to antigen presentation and energy metabolism have been discovered, such as calreticulin and adenylate kinase 2 (AK2) (Wilson et al., 2013). Recent study findings have revealed that DPP8/9 cleavage sites exist in more than 100 mitochondrial proteins including AK2, and DPP8/9 can impact the intracellular levels of these proteins (Finger et al., 2020). Since AK2 plays an essential role in maintaining cellular energy homeostasis, DPP8 and DPP9 may regulate energy metabolism by cleaving AK2.

Although the effect of DPP8/9 cleavage on the function of these substrates remains to be further investigated, their identification highlights the potential role of DPP8/9 in immune regulation and energy metabolism.

## 3 Biological activity and physiological functions of DPP8/9

### 3.1 DPP8/9 and the maintenance of normal development and physiological functions

Gall, M.G, Kim, M, et al. scholars constructed a mouse model of DPP9 enzyme inactivation. The gene knock-in (gki) mice resulted in the loss of enzymatic activity due to a serine to alanine point mutation (S729A) in the active site of the DPP9 enzyme. Increased apoptosis in occipital somite-derived migratory muscle progenitors in homozygous *Dpp9^ki/ki^* mice resulted in abnormal formation of intrinsic muscles at the distal tongue. Microglossia causes sucking defects in neonates that die within 24 h of birth. The neonates can survive manual feeding (Gall et al., 2013; Kim et al., 2017). A new study also showed that deletion of *Nlrp1* and its downstream genes such as *Asc*, *Gsdmd*, and *Il-1r* could reduce neonatal death in *Dpp9^ki/ki^* mice. It indicated that DPP9 enzymatic inactivation could function through the NLRP1 signaling pathway (Harapas et al., 2021). In addition, neonatal DPP9-enzyme-inactivated mice showed differences in the expression of genes related to cell growth, innate immunity, and metabolism in the liver and gut compared with wild-type mice (Chen et al., 2016). However, inactivation of DPP9 enzymatic activity in mice does not significantly affect hematopoiesis and immune regeneration (Kim et al., 2018; Gall et al., 2019). The nonselective inhibitor of DPP4 saxagliptin has also been shown to affect myocardial electrophysiology [Ca^2+^ transient relaxation impaired and action potential duration (APD) prolonged] by inhibiting the enzymatic activity of DPP9 (Koyani et al., 2018). And altered methylation of the *DPP9* gene is associated with human cardiac dysfunction (Nguyen et al., 2022).

DPP8/9 is involved in cell adhesion and migration. Previous studies showed that DPP9 contains an Arg-Gly-Asp sequence that could bind to integrins (Ajami et al., 2004). Overexpression of DPP8/9 could impair the interaction of HEK293T cells with the extracellular matrix, and inhibit adhesion and migration *in vitro*, an effect that was independent of the enzymatic activity of DPP8/9 (Yu et al., 2006). In human skin cells, however, DPP9 could be located at the edge of the plasma membrane of fibroblasts and keratinocytes, and *DPP9* gene silencing resulted in reduced cell adhesion and migration (Gabrilovac et al., 2017). A recent study also indicated that the levels of adenylyl cyclase-associated protein 1 (CAP1), a protein that regulated cellular actin dynamics and cytoskeleton remodeling, were altered after treatment of macrophages with DPP8/9 selective inhibitor 1G244. The expression of talin was also down-regulated (Suski et al., 2020). In addition, some studies have found that DPP9 is involved in the adhesion and migration of tumor cells. For example, in tumor cells Huh7 and HeLa, the localization of DPP9 was found to be associated with mitochondria and microtubules. DPP9 was redistributed towards the ruffling membrane upon stimulation, where it colocalized with cell adhesion-related focal adhesion protein, integrin-β1, and talin. When the expression and activity of DPP9 were inhibited, cell adhesion and migration were also reduced, the expression of integrin-β1 and talin was reduced, and the phosphorylation levels of adhesion-focal kinase and paxillin were decreased (Zhang et al., 2015b). In a study of non-small cell lung cancer, it was found that inhibition of DPP9 expression in A549 and H1299 cell lines reduced cell migration and invasion (Tang et al., 2017). However, overexpression of DPP9 in human oral squamous cell carcinoma (OSCC) cells could reduce cell migration, invasion, and adhesion (Wu et al., 2020). Nucleobindin-1 (NUCB1), a substrate of DPP9, was also found to bind to matrix metalloproteinase 2 (MMP2) in a Ca^2+^-dependent manner, which can degrade extracellular matrix (ECM) proteins. The interaction between NUCB1 and MMP2 was involved in the transport of MMP2 in the Golgi (Pacheco-Fernandez et al., 2020). These findings suggest that DPP9 may be involved in tumor metastasis. Moreover, it was found that knockdown of *DPP8* in HeLa and SiHa cells also inhibited the expression of MMP2 and MMP9, reducing cell migration and invasion (Chen et al., 2018).

DPP8/9 is also involved in cellular energy metabolism. As aforementioned, the expression of metabolism-related genes and the levels of metabolic-related products were altered in the liver and gut of mouse neonates with inactive DPP9 enzymatic activity, resulting in dysregulation of lipid metabolism and glucose metabolism. The *Dpp9* knockdown in liver cancer cells mediated the energy catabolism pathways by increasing the activation of AMPK, a regulator of anabolism (Chen et al., 2016). Wilson et al. (2013) identified some DPP8/9 substrates related to cellular metabolism and energy homeostasis. For example, AK2 involved in the regulation of cellular energy balance is a potential substrate of DPP8/9. Some substrates of DPP8 are mature mitochondrial proteins, such as acetyl-CoA acetyltransferase. Recent studies have identified more than 100 mitochondrial proteins as potential substrates of DPP8/9, including AK2 as well. DPP8/9 can process the N-terminus of AK2. The processed AK2 could be accelerated for degradation by the proteasome, thereby reducing the accumulation of enzymatically active AK2 in the cytoplasm (Finger et al., 2020). The use of DPP8/9 selective inhibitor 1G244 or knockdown of *Dpp8/9* in preadipocytes resulted in impaired adipocyte differentiation, whereas the use of PPARγ2 agonists or PPARγ2 overexpression restored preadipocyte differentiation. Therefore, DPP8 and DPP9 may participate in adipogenesis through the PPARγ2 signaling pathway (Han et al., 2015). Administration of DPP8/9 inhibitors prior to activation of THP-1 macrophages altered the levels of metabolism- and mitochondria-related proteins in cells, the downregulation of key enzymes in some signaling pathways such as glycolysis, pentose phosphate pathway (PPP), and hypoxia-inducible factor-1 (HIF-1). DPP8/9 inhibitors also affected some markers that maintain mitochondrial structural integrity and function (Suski et al., 2020). The *Dpp9* gene has also been found to be associated with feline diabetes mellitus (Forcada et al., 2021).

Additionally, DPP8/9 is also involved in cell proliferation and apoptosis. Studies have shown that the use of DPP8/9 inhibitors and *DPP9* silencing can inhibit the proliferation of some cells, such as activated T cells (Lankas et al., 2005; Reinhold et al., 2009), fibroblasts, HaCaT cells (Gabrilovac et al., 2017), and macrophage cells (Zapletal et al., 2017). In the study of atherosclerosis, DPP8/9 inhibitors can promote macrophage apoptosis (Matheeussen et al., 2013). Administration of DPP8/9 inhibitors before THP-1 macrophage activation can also alter the levels of apoptosis-related proteins involved in intracellular and extracellular (Suski et al., 2020). In contrast, overexpression of *DPP9* in HEK293 cells induced apoptosis, and DPP9 enzyme-negative mutants also exhibited the same effect, so the enzyme activity might not be necessary to induce apoptosis in HEK293 cells (Yu et al., 2006). Moreover, DPP8/9 is also involved in the regulation of some tumor cell proliferation and apoptosis, which will be discussed in the tumor section.

### 3.2 DPP8/9 and immune regulation

Numerous studies have shown that DPP8/9 is involved in the regulation of the immune system. In humans and animals, DPP8 and DPP9 have been detected to be widely expressed in the immune system, such as lymphocytes (Abbott et al., 2000; Olsen and Wagtmann, 2002; Maes et al., 2007; Yu et al., 2009; Chowdhury et al., 2013), monocytes (Maes et al., 2007), and macrophage cells (Zapletal et al., 2017), indicating that DPP8 and DPP9 may play an immunoregulatory function in these cells. And as previously described, the expression levels of immune-related genes were altered in Dpp9^ki/ki^ mice (Chen et al., 2016).

In earlier studies on DPP4, nonselective inhibitors of DPP4 were found to inhibit DNA synthesis in natural killer (NK) cells and B lymphocytes (Buhling et al., 1994; Buhling et al., 1995). These inhibitors were later shown to have inhibitory effects on DPP8/9, as such, inhibition of the enzymatic activity of DPP8/9 may also play a role. DPP8/9 is widely expressed in lymphocytes, and expression is up-regulated in activated lymphocytes, suggesting that DPP8/9 may be involved in lymphocyte activation (Chowdhury et al., 2013). In wild-type C57BL/6 mice and DPP4-knockout mice, DPP4 selective inhibitors could not inhibit the activation of mouse splenocytes, while nonselective inhibitors for DPP4 and selective inhibitors for DPP8/9 could inhibit DNA synthesis within stimulated splenocytes (Reinhold et al., 2009), indicating that DPP8/9 is involved in the activation of immune cells rather than DPP4. DPP8/9 inhibitors could also inhibit the activation of human peripheral blood T cells *in vitro* (Ansorge et al., 2011). Additionally, inhibition of DPP8/9 could attenuate the activation of macrophages (Matheeussen et al., 2013; Waumans et al., 2016).

Zapletal et al. (2017) showed that the expression of intracellular DPP9 was decreased after activation of mouse macrophage-derived J774 cell line with interferon γ (IFN‐γ) and lipopolysaccharide (LPS). After silencing the *Dpp9* gene in inactivated cells, the activation marker C‐C chemokine receptor type 5 (CCR5) also increased correspondingly, indicating that DPP9 possibly inhibits the activation of macrophages. It is in contrast to the above conclusion that DPP9 exerts a pro-inflammatory role, which the authors suggest may be due to the different methods of assessing macrophage activation, namely the difference between the two assessments of CCR5 expression and secretion of pro-inflammatory cytokines.

In addition, DPP9 may also be involved in antigen presentation. The enzymatic activity of DPP9 was proved to be rate-limiting in the degradation of proline-containing antigenic peptides (Geiss-Friedlander et al., 2009). And in the process of endocytosis, DPP9 is located in organelles/vesicles, which may be involved in antigen peptide processing (Zapletal et al., 2017).

## 4 DPP8/9 and diseases

### 4.1 DPP8/9 and tumors

DPP8/9 has been confirmed to be expressed in a variety of different types of tumors, such as brain tumors, gynecological malignancies, liver cancer, and hematological malignant tumors. By searching the human protein atlas, DPP9 was tagged as a prognostic marker for multiple tumors (The Human Protein Atlas, 2022). However, the specific mechanism of DPP8/9 in tumorigenesis and development is still being explored. Studies have shown that DPP8/9 may participate in the proliferation, apoptosis, migration, and invasion of tumor cells. Nonselective inhibitors of the DPPs family can exert anti-tumor effects by regulating immunity, such as regulating cytokines, chemokines, and immune cells (such as T cells and dendritic cells) to inhibit tumor growth (Adams et al., 2004; Duncan et al., 2013; Walsh et al., 2013; Henderson et al., 2021).

It has been implicated that DPP8/9 is expressed in various brain tumors, such as gliomas and meningiomas (Sedo et al., 2004; Stremenova et al., 2007; Stremenova et al., 2010; Busek et al., 2012). In gliomas, DPP4-like enzyme activity increases with tumor more malignancy, mainly due to DPP4 and FAP (Stremenova et al., 2007). Non-malignant brain tissue contains DPP4-like enzymatic activity primarily attributable to DPP8/9 (Stremenova et al., 2007), it has also been shown that DPP9 is upregulated in glioma (Zheng et al., 2022). In contrast, more abundant expression of DPP8/9 was currently detected in meningiomas. DPP4-like enzymatic activity is mainly derived from DPP8/9 (Stremenova et al., 2010).

Studies have shown that DPP8/9 plays a role in hematological tumors. Abnormal upregulation of DPP8/9 expression can be detected in B-cell chronic lymphocytic leukemia (CLL) (Sulda et al., 2010). The DPP8/9 expression in Jurkat T and Raji B cell lines upon stimulation is altered, affecting apoptosis (Chowdhury et al., 2013). Clinical trials of the combination of the DPP4 nonselective inhibitor Val-boro-Pro and rituximab in the treatment of primary drug-resistant and indolent non-Hodgkin’s lymphoma and chronic lymphocytic leukemia (CLL) have been conducted in the early years (Al-Katib et al., 2004; Khan et al., 2005). Val-boro-Pro can enhance the ability of rituximab and trastuzumab against CD20^+^ B-cell lymphoma via immunomodulation (Adams et al., 2004). The DPP4 inhibitor vildagliptin was found to coordinately enhance the toxicity of parthenolide to leukemia cells by inhibiting DPP8/9 (Spagnuolo et al., 2013). DPP8/9 inhibitors have been implied to induce pyroptosis in human acute myeloid leukemia (AML) cell lines and primary AML samples, they have been validated in mouse models as well (Johnson et al., 2018). In recent years, DPP8/9 has made significant progress in the research on the mechanism of pyroptosis of blood system cells, and DPP8/9 inhibitors can be used as a potential therapeutic drug for AML. In multiple myeloma, inhibition of DPP8 activity and expression can exert anti-tumor effects by inducing apoptosis (Sato et al., 2019).

In studies of Gynecologic malignancies, DPP8/9 was indicated to be ubiquitous in breast cancer, ovarian cancer, and HeLa cell lines (Wilson and Abbott, 2012). It was revealed that DPP8/9 interacting with H-Ras in HeLa cells (Yao et al., 2011), and inhibiting the expression and activity of DPP9 in HeLa cells could reduce the adhesion and migration ability of cells (Zhang et al., 2015b). In ovarian clear cell adenocarcinoma, *DPP9* was identified as a gene with copy number aberrations (Sung et al., 2013). In high-grade serous ovarian cancer, *DPP9* was involved in two gene rearrangements (rearrangement of *DPP9* with *PPP6R3* and *DPP9* with *PLIN3* respectively), and the expression of *DPP9* 3′ end was reduced, resulting in DPP9 losing the active domains (Smebye et al., 2017). *DPP9* loss-of-function (LoF) variants were also frequently associated with endometrial, cervical, or ovarian carcinoma (Huang et al., 2021b). Elevated DPP8/9 levels were also detected in ovarian carcinoma and their exudates. The expression of DPP8/9 was enhanced in aggressive and metastatic tumors, suggesting that DPP8/9 might be involved in tumor progression (Brunetti et al., 2019). CXCL10, a substrate of DPP8/9, was also found to play an important role in ovarian carcinoma by immune regulation (Rainczuk et al., 2014). In breast cancer, DPP9 expression was elevated, and high DPP8/9 expression correlates with a good prognosis (Choy et al., 2021). However, it has also been reported that DPP8 promoted breast tumor growth (Huo et al., 2016). And DPP9 was found to be involved in the metastasis of breast cancer cells (Sui et al., 2022). Moreover, DPP8 has also been indicated to participate in the pathogenesis of cervical cancer (Huo et al., 2016). After silencing *DPP8* in human cervical cancer cell lines, G1 phase arrest increased Bax expression, decreased BCL2 expression, inhibited cell proliferation, promoted cell apoptosis, and inhibited the expression of MMP2 and MMP9, reducing cell migration and invasion (Chen et al., 2018). The expression of DPP8/9 was also detected in the reproductive system of male animals, but the specific role is not clear so far. The *DPP9* gene is significantly upregulated in human testicular tumors (Yu et al., 2009). Among human prostate cancer cell lines, the highly invasive cell lines PC3 and DU145 have higher DPP9 activity (Nomura et al., 2011).

Overexpressed DPP8/9 in patients with hepatocellular carcinoma (HCC) has been observed (Huang et al., 2021b). DPP9 in human liver cancer cells (HepG2 and Huh7) could promote apoptosis and reduce proliferation by inhibiting the EGFR/PI3K/Akt signaling pathway (Yao et al., 2011; Chowdhury et al., 2013), and inhibiting the expression and activity of DPP9 in Huh7 cells could decrease the adhesion and migration ability of cells (Zhang et al., 2015b). The level of DPP9 was higher in lesions of a mouse model of HCC than that in surrounding normal tissue. The DPP4 family inhibitor ARI-4175 could increase the activation of caspase-1 and the infiltration of CD8^+^ T cells in the liver, resulting in a decrease in the number of liver nodules (Henderson et al., 2021). A report in 2022 has shown that breast cancer-associated protein 2 (BRCA2), a substrate of DPP9, plays an essential role in a variety of tumors, including HCC and breast cancer. DPP9 can affect cellular concentration of BRCA2 by modifying the N-terminus of BRCA2, which may regulate the interaction between BRCA2 and RAD51, and further promote damaged DNA repair (Bolgi et al., 2022).

Besides, in non-small cell lung cancer (NSCLC), DPP9 was involved in epithelial-mesenchymal transition, and inhibition of DPP9 expression inhibited cell proliferation, migration, and invasion, and promotes apoptosis (Tang et al., 2017). And in lung adenocarcinoma samples, CircD-DPP9 was identified as a reduced extrachromosomal circular DNA (eccDNA) (Xu et al., 2022). In Ewing’s sarcoma, DPPs could prevent cell death by cleaving (neuropeptide Y) NPY, and inhibition of DPP8/9 can activate poly (ADP-ribose) polymerase (PARP-1) and apoptosis-inducing factor (AIF)-mediated apoptosis pathway (Lu et al., 2011). DPP9 is also considered to be a poor prognostic factor for metastatic colorectal cancer, and inhibiting the activity or expression of DPP9 can inhibit the viability of tumor cells and increase the sensitivity to antitumor drugs (Saso et al., 2020). Conversely, studies have also shown that DPP9 expression is reduced in human oral squamous cell carcinoma (OSCC), knockout of *DPP9* in tumor cells accelerates cell proliferation, while overexpression of *DPP9* inhibits tumor cell proliferation, invasion, metastasis, and epithelial-mesenchymal transition (Wu et al., 2020).

In summary, DPP8/9 is involved in the occurrence and development of various tumors and play different roles in different tumor types and tumor stages. Recent studies have further clarified this point of view. It has shown that the abnormal expression and enzymatic activity of DPP8/9 could participate in the occurrence and development of tumors by affecting cell proliferation and apoptosis, adhesion and migration, damaged DNA repair, and immune regulation. However, the underlying mechanisms and whether these tumors are possibly treated through regulating the enzyme activity and expression of DPP8/9 remain unclear, that need to be further explored.

### 4.2 DPP8/9 and inflammation

#### 4.2.1 Inflammation

Previous studies have shown that DPP4 is involved in the induction of inflammatory responses (Kruschinski et al., 2005; Ohnuma et al., 2008). As members of the DPP4 family, DPP8/9 may also be involved in the inflammatory response, and a large number of studies have proved this point.

The relationship between DPP4 and rheumatoid arthritis was investigated in rat models of arthritis induced by type II collagen or alkyldiamine, and the induction of arthritis could be inhibited by using DPP4 inhibitors (Tanaka et al., 1997; Tanaka et al., 1998). But reported DPP4 inhibitors were less specific; it was further found that induced arthritis persisted and was more severe in DPP4-deficient mice (Busso et al., 2005), so it may play an anti-inflammatory role by inhibiting other members of the DPP4 family, such as DPP8/9.

In a rat asthma model induced by sensitization and challenge with ovalbumin, the gene level and enzymatic activity of DPP8/9 in lung tissue of wild-type and DPP4-knockout rats and the enzymatic activity of DPP8/9 in bronchial lavage fluid were upregulated. Therefore, DPP8 and DPP9 may be involved in the occurrence and development of chronic inflammatory responses in asthma (Schade et al., 2008). In asthmatic patients carrying the NLRP1 variant M1184V (methionine 1,184 to valine), inhibition of DPP9 enzymatic activity reduces inflammasome activation, possibly exacerbating asthma (Moecking et al., 2020). In the rat model of neuroinflammation induced by cerebral ischemia, the expression and activity of the DPP4 family in cortical neurons, microglia, and macrophages changed at different time points, including DPP8/9 (Rohnert et al., 2012). DPP4 has been shown to play an important role in the chemically induced Crohn’s disease (CD) mouse model. After *Dpp4* was knocked out, the expression and activity of DPP8 and DPP9 in the colon of mice changed at different stages of the disease. The concentration and enzymatic activity of DPP8/9 were also altered in the serum of mice (Buljevic et al., 2018). And in the colorectal tissue of patients with inflammatory bowel disease (IBD), the expression and enzyme activity of DPP8/9 were found to be increased (Jaenisch et al., 2022), which indicated that DPP8/9 may be involved in the development of the disease and healing process.

The expression of DPPs was examined in atherosclerotic plaques and the presence of DPP8/9 was detected in the macrophage region. DPP9 expression was also elevated during monocyte-to-macrophage differentiation. The activation of M1-like macrophages was inhibited using the DPP8/9 inhibitor 1G244, and silencing *DPP9* expression reduced the secretion of inflammatory factors such as TNFα and IL-6, indicating that DPP9 plays an important role in chronic inflammation (Matheeussen et al., 2013). This effect was also validated in a mouse model of atherosclerosis (Waumans et al., 2016). Recently, it was found by the proteomic method that prior to the activation of THP-1 macrophages, DPP8/9 inhibitors inhibited the activation of M1-like macrophages, and reduced the level of markers of macrophage activation and inflammatory factors secreted by macrophages. In the meantime, the levels of some proteins related to endoplasmic reticulum stress were also regulated, and these proteins might also be involved in the inflammatory response (Suski et al., 2020).

#### 4.2.2 DPP8/9 and pyroptosis

The inflammasome can exert pro-inflammatory effects to stimulate the immune system by activating caspase-1 to trigger a lytic form of programmed cell death known as pyroptosis. In recent years, DPP8/9 inhibitors have been discovered to induce this process.

**FIGURE 2:**
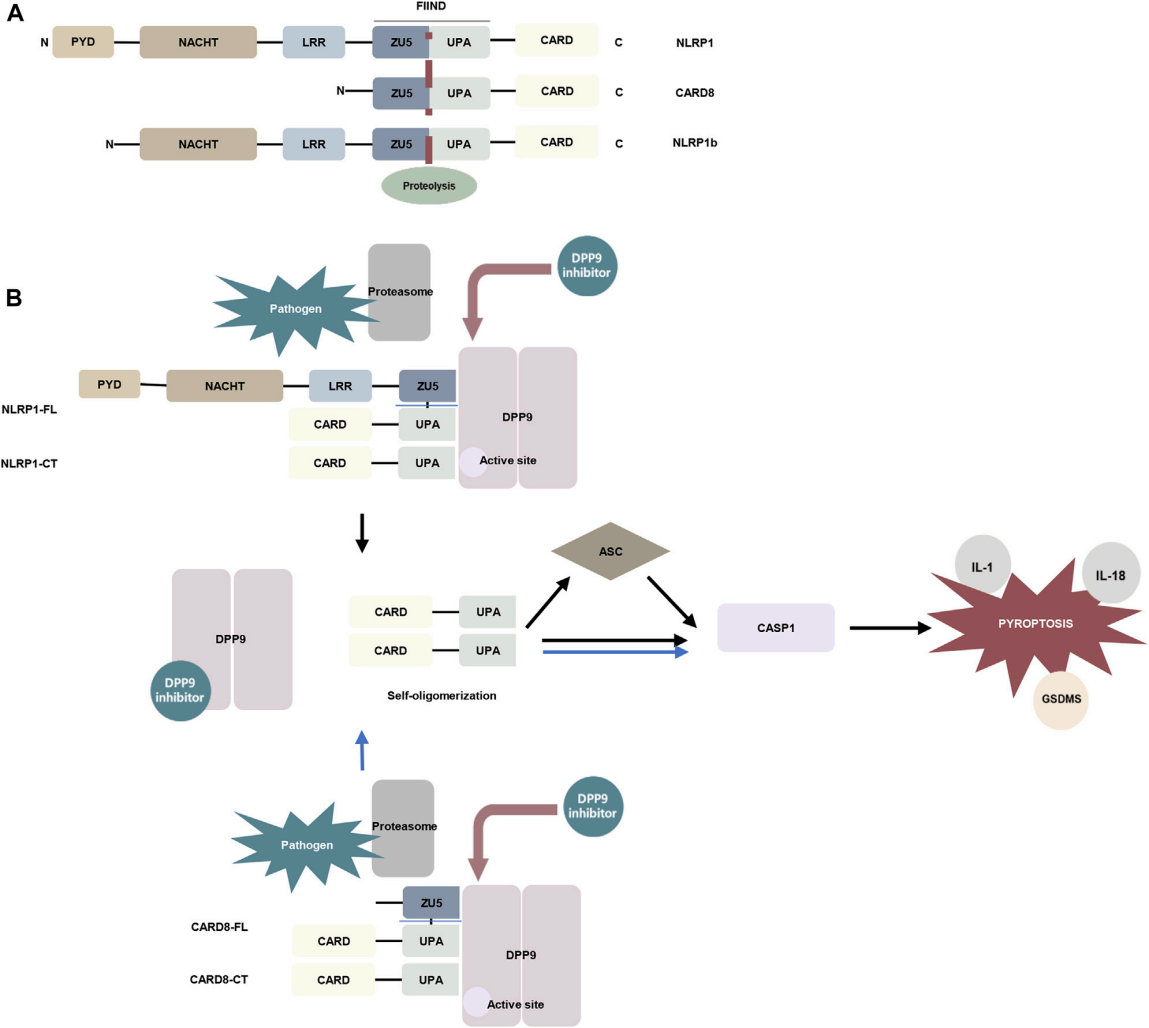
The mechanism of DPP9 is involved in pyroptosis. **(A)** Schematic representation of the domains of human NLRP1, human CARD8, and mouse NLRP1b. In the FIIND domain, the ZU5 and UPA subdomains can directly undergo autoproteolysis to generate the active fragment of UPA-CARD (Frew et al., 2012; D'Osualdo et al., 2011). **(B)** DPP9 forms a 1:2 ternary complex with NLRP1(NLRP1-FL and NLRP1-CT) through the FIIND domain to inhibit the activity of NLRP1, and NLRP1-CT inserts into the DPP9 active site (Huang et al., 2021a; Hollingsworth et al., 2021). Pathogen-induced proteasome degradation of the N-terminus of NLRP1 can result in the release of active UPA-CARD fragments from the complex (Gong et al., 2021; Robert Hollingsworth et al., 2021). DPP8/9 inhibitors can attenuate the interaction of DPP9 with NLRP1. The released UPA-CARD fragments oligomerize and recruit ASC and caspase-1, which in turn triggers pyroptosis. The DPP9-CARD8 interaction is similar to NLRP1, but CARD8-CT does not interact with the DPP9 active site (Sharif et al., 2021). (Abbreviation: PYD, pyrin domain; NACHT, nucleotide-binding; LRR, leucine-rich repeat; ZU5, ZO-1 and UNC5; UPA, UNC5, PIDD, and Ankyrin; CARD, caspase activation and recruitment domain; ASC, apoptosis-associated speck-like protein containing a CARD; GSDMS, gasdermin D).

Inflammasomes can be formed from the nucleotide-binding domain leucine-rich repeat (NLR) family of proteins. NLRP1 and the human-specific inflammatory regulator CARD8 are the only human proteins that contain an autoproteolytic domain named function-to-find (FIIND) domain. The FIIND domain consists of ZU5 and UPA subdomains. CARD8 is similar to the C-terminal region of NLRP1 and contains the FIIND domain and caspase activation and recruitment (CARD) domain. Nlrp1b is a homolog of NLRP1 and CARD8 (Frew et al., 2012) (Figure 2A). NLRP1 and CARD8 can undergo autoproteolysis between the ZU5 and UPA domains, releasing the UPA–CARD fragment, which in turn activates caspase-1 (D'Osualdo et al., 2011; Gong et al., 2021; Robert Hollingsworth et al., 2021).

The DPP family nonselective inhibitor Val-boroPro and the DPP8/9 selective inhibitor 1G244 were indicated to induce pyroptosis in mouse monocytes and macrophages (Okondo et al., 2017). Inhibition of DPP8/9 causes autoproteolysis of the FIIND domain of the inflammasome sensor protein Nlrp1b to activate Nlrp1b, which in turn activates caspase-1 to induce pyroptosis by cleaving gasdermin D (GSDMD). And this process is independent of the inflammasome adaptor ASC (Okondo et al., 2017; Taabazuing et al., 2017; Okondo et al., 2018). Nevertheless, in murine macrophages expressing *Bacillus anthracis* lethal toxin (LeTx)–sensitive *Nlrp1b* allele, Nlrp1b inflammasome activation upon DPP8/9 inhibition promotes ASC speck assembly, mediates caspase-1 automaturation, and triggers accelerated pyroptosis. Deletion of ASC attenuates caspase-1 activation and pyroptosis (de Vasconcelos et al., 2019). These findings have also been confirmed *in vivo*. Deletion of NLRP1 and its signaling downstream molecules ASC, GSDMD, and IL-1 allowed the survival of mice with DPP9 enzyme inactivation (Harapas et al., 2021). Subsequent studies have found that DPP8/9 inhibitors can activate all functional rodent *Nlrp1b* alleles, including mouse *Nlrp1a*, three functional *Nlrp1b* alleles, and all rat *Nlrp1* alleles (Gai et al., 2019). Rat DPP9 forms a 1:2 complex with rat NLRP1, which contains a full-length NLRP1 and a UPA-CARD fragment of NLRP1 (Figure 2B) (Huang et al., 2021a).

Inhibition of DPP8/9 can also induce pyroptosis in human cells. DPP8/9 inhibitor induces pyroptosis through CARD8 activation of pro-caspase-1 demonstrated in human acute myeloid leukemia (AML) cell lines and primary AML samples (Johnson et al., 2018). Two families carrying rare *DPP9* variants with clinical manifestations associated with Autoinflammation with Arthritis and Dyskeratosis (AIADK) have been recently reported. AIADK is a disease caused by dysregulation of the NLRP1 inflammasome. *DPP9* variants have been found to reduce DPP9 expression and enzymatic activity in primary skin cells isolated from patients in these two families and finally activate NLRP1 (Harapas et al., 2021). In human keratinocytes and monocytes, DPP9 was shown to interact with the FIIND domains of NLRP1 and CARD8. DPP9 can maintain the inactive state of NLRP1, which depends on the enzymatic activity of DPP9 (Zhong et al., 2018). The human NLRP1–DPP9 complex was found to be similar in structure to the rat complex described above, a ternary complex consisting of DPP9, full-length NLRP1 (NLRP1-FL), and the inflammatory C-terminal fragment of NLRP1 (NLRP1-CT), in which the NLRP1-CT was inserted into the DPP9 active site (Figure 2B). DPP8/9 inhibitors or deletion of DPP9 disrupt the DPP9-NLRP1 interaction to induce pyroptosis (Zhong et al., 2018; Hollingsworth et al., 2021). Whereas NPRP1 M1184V enhanced its binding to DPP9 and decreased inflammasome activation after DPP9 inhibition (Moecking et al., 2020). The DPP9-CARD8 interaction is similar to NLRP1 and consists of DPP9, full-length CARD8 (CARD8-FL), and a C-terminal fragment of CARD8 (CARD8-CT) to form a stable ternary complex. The difference is that CARD8-CT does not interact with the DPP9 active site (Sharif et al., 2021) (Figure 2B). This notion was also supported by an earlier study showing that the DPP9-CARD8 interaction is not blocked by DPP8/9 inhibitors (Griswold et al., 2019). And DPP8/9 inhibitors can activate a proteasomal degradation pathway that destroys disordered and misfolded proteins. Because the N-terminal of CARD8 contains a disordered region, it can be recognized and disrupted by this pathway and in turn, release its C-terminal fragment to induce pyroptosis (Chui et al., 2020). Moreover, Val-boroPro has also been shown to induce pyroptosis in human and rodent resting but not activated lymphocytes. In human resting T lymphocytes, DPP9 is involved in cell pyroptosis through the CARD8-CASP1-GSDMD signaling axis (Johnson et al., 2020; Linder et al., 2020).

In conclusion, recent studies have shown that DPP8/9 plays an essential role in maintaining the stability of NLRP1 and CARD8 and inhibiting pyroptosis. DPP8/9 inhibitors have the potential to treat hematological diseases, but their potential side effects, such as induction of resting lymphocytes pyroptosis, are still unclear. Their mechanism of function and possible clinical application needs to be further investigated.

### 4.3 DPP8/9 and organ fibrosis

In an animal model of early hepatic fibrosis induced by carbon tetrachloride (CCl_4_), intrahepatic DPP8/9 expression was increased. Conversely, the expression is downregulated in end-stage human chronic liver injury [such as advanced primary biliary cirrhosis (PBC) and end-stage alcoholic liver disease (ALD)] and the animal model of primary sclerosing cholangitis, the *Mdr2* gko mouse (Yao et al., 2011; Chowdhury et al., 2013). Therefore, the expression of DPP8/9 is associated with the pathological type of liver disease. They are upregulated in the acute phase of the disease and downregulated in the chronic phase and may be involved in the development of liver diseases, but their underlying mechanisms remain elusive. Furthermore, the *DPP9* gene was linked to idiopathic pulmonary fibrosis by genome-wide association studies (GWAS) (Fingerlin et al., 2013; Hobbs et al., 2019).

Our previous study results revealed that DPP8/9 expression was increased in proximal tubular epithelial cells in patients with chronic kidney disease (CKD). It has been demonstrated that DPP8/9 can affect the transforming growth factor β1 (TGF-β1)/Smad signaling pathway using the CKD animal models induced by unilateral ureter obstruction and cell culture studies respectively. Administration of DPP8/9 inhibitor or *DPP8/9* silencing could improve the occurrence and development of kidney inflammation and fibrosis, indicating that DPP8/9 may be a potential therapeutic target for tubulointerstitial fibrosis (Zhang et al., 2021).

These research findings suggest that DPP8/9 may be a very important target for the treatment of organ fibrosis. However, there are few relevant experimental data and a lack of animal safety reports on the long-term DPP8/9 selective inhibitors application. Therefore, the more experimental evidence about the regulation of DPP8/9 expression and enzymatic activity for the treatment of organ fibrosis is needed.

### 4.4 DPP8/9 and COVID-19

Studies implicated the *DPP9* gene was involved in Coronavirus disease 2019 (COVID-19) caused by severe acute respiratory syndrome coronavirus 2 (SARS-CoV-2) (Pairo-Castineira et al., 2021; Wang et al., 2021; Horowitz et al., 2022; Sharif-Zak et al., 2022). As aforementioned, DPP9 is associated with pulmonary fibrosis, which is a hallmark of critically ill patients with COVID-19. The *DPP9* gene correlated with the severity of the disease, with higher levels of DPP9 in the peripheral blood of critically ill patients (Wang et al., 2021; Horowitz et al., 2022; Sharif-Zak et al., 2022). Therefore, DPP9 could be used as a potential risk assessment indicator for COVID-19 patients. However, the possible role of DPP9 in COVID-19 remains unclear. The expression level of DPP9 in COVID-19 male patients was higher than that in female patients (Sharif-Zak et al., 2022), and the expression of DPP9 was correlated with gonadal steroids (Mayneris-Perxachs et al., 2021), suggesting that gender might be an important influencing factor. DPP9 might function by affecting the activation of inflammasomes. In critically ill patients with COVID-19, high levels of DPP9 may act to suppress inflammation (Wang et al., 2021; Sharif-Zak et al., 2022). In addition, DPP9 is upregulated in chronic alcoholic brain tissue (Muhammad et al., 2021) and downregulated in UVB-irradiated human cells, so alcohol avoidance and appropriate sunlight exposure may be beneficial for COVID-19 infection by regulating the DPP9 expression. And through lipidomics, DPP9 was found to be linked to some fatty acids, which provided evidence to modulate inflammation in patients through diet (Mayneris-Perxachs et al., 2021). Further research on the mechanism of action of DPP9 will contribute to the potential prevention and treatment of COVID-19.

## 5 Research progress of DPP8/9 inhibitors

DPP8/9 exerts biological functions by cleaving their substrates, so the development of selective inhibitors is of great significance to explore the mechanisms of action of DPP8/9 and their potential future use in the treatment of diseases. 1G244 and Allo-Ile-isoindoline are the most common highly selective inhibitors of DPP8/9, both of them are derivatives of isoindoline (Jiaang et al., 2005; Lankas et al., 2005). Allo-Ile-isoindoline is a competitive inhibitor of DPP8/9, but its membrane penetration is poor and its inhibitory effect on DPP8/9 in the cytoplasm is weak (Wu et al., 2009; Bank et al., 2011), the serious toxicity on cell culture and animal models in previous studies were observed, such as alopecia, thrombocytopenia, reticulocytopenia, increased mortality in animals, and attenuated human T cell activation *in vitro* (Lankas et al., 2005), which may be attributed to off-target effects rather than inhibition of DPP8/9 enzymatic activity. 1G244 is an irreversible inhibitor of DPP8 (a slow and tight-binding competitive inhibitor) and a competitive inhibitor of DPP9 (Wu et al., 2009), but in the study of the molecular crystal structure, it was found that the two were synergistic in binding with 1G244 (Ross et al., 2018). Compared with Allo-Ile-isoindoline, 1G244 was characterized by better membrane penetration and higher potency, and the aforementioned adverse reactions did not occur in animal experiments (Wu et al., 2009), which made DPP8/9 inhibitors the potential drugs to treat diseases with existing DPP8/9 abnormalities. However, as mentioned earlier, recent research results have revealed that DPP8/9 inhibitors cause the pyroptosis of resting T cells (Johnson et al., 2020; Linder et al., 2020). In addition, many groups have constructed their analogs with high selectivity to DPP8/9 on the basis of Allo-Ile-isoindoline and 1G244, such as Allo-Ile-isoindoline analogs (8j) (Van der Veken et al., 2008; Van Goethem et al., 2008) and 1G244 analogs (Van Goethem et al., 2011). Because the sequences and structures of DPP8 and DPP9 are very similar, there are no selective inhibitors for DPP8 or DPP9 respectively available now. The methylpiperazine analogs of 1G244 constructed by Van Goethem et al. (2011) team and the isoindoline-derived molecules constructed by Benramdane et al. (2022) have a certain selectivity, which provides a direction for selective inhibitor design. However, DPP8 and DPP9 were found to have consistent allosteric interactions with their substrates in molecular dynamics simulations, and the authors believed that it was difficult to design targeting ligands that distinguish the two (Ross et al., 2018). Furthermore, the aforementioned SUMO1-EIL peptide variant, SLRFLYEG, is a potent selective allosteric inhibitor (Pilla et al., 2013) (Table 2).

**TABLE 2:** Potency of selective DPP8/9 inhibitors.

DPP8/9 inhibitors	IC50(nM)		Ki(nM)		References
	DPP8	DPP9	DPP8	DPP9	
Allo-Ile-isoindoline	38	55			Lankas et al. (2005)
	120 ± 10	290 ± 20			Van der Veken et al. (2008), Van Goethem et al. (2008)
	145	242	13.7	33.7	Wu et al. (2009)
1G244	14	53	0.9	4.2	Jiaang et al. (2005), Wu et al. (2009)
	12 ± 1	84 ± 2			Van Goethem et al. (2011)
Methylpiperazine analogues of 1G244 (compound 12m)	32 ± 2	260 ± 20			Van Goethem et al. (2011)
Methylpiperazine analogues of 1G244 (compound 12n)	50 ± 5	540 ± 40			
compound 2e (Irreversible inhibitor)	520 ± 110				Van der Veken et al. (2007)
Analogue of allo-Ile-isoindoline (compound 8j)	160 ± 16	70 ± 40			Van Goethem et al. (2008)
Isoindoline-derived molecule (compound 5s)	3,100 ± 100	490 ± 40			Benramdane et al. (2022)
Isoindoline-derived molecule (compound 5u)	3,300 ± 500	500 ± 200			
SUMO1-EIL peptide (SLRFLYEG)			147 ± 11.13	170 ± 11.28	Pilla et al. (2013)

## 6 Discussion

DPP8 and DPP9 belong to the serine protease family and are ubiquitously expressed in animals and humans. Although they are very similar in structure and sequence, their functions are not completely identical. Since the substrates and interaction partners of DPP8/9 are still unclear, and unlike the well-known localization of DPP4, they are mainly localized in cells, their biological functions require further studies to investigate. Previous studies have shown that DPP8 and DPP9 are multifunctional proteins that can function through enzymatic and non-enzymatic activities and participate in various physiological and pathological processes. In this review, we summarize the known functions of DPP8/9, which are involved in biological processes such as cell adhesion and migration, cell energy metabolism, cell proliferation and apoptosis, and immune regulation. And they play a role in pathological processes such as tumorigenesis, inflammation, and fibrosis. DPP8/9 is expected to be a potential therapeutic target for these relevant diseases.

Studies in recent years have further proved that DPP8/9 is involved in the occurrence and development of various tumors, and their potential mechanisms have been explored. For example, inhibition of DPP8 expression in cervical cancer cells can inhibit cell proliferation, migration, and invasion by affecting the expression of Bax and BCL2, inhibiting the expression of MMP2 and MMP9 (Chen et al., 2018). DPP9 affects the adhesive migration of tumor cells via colocalizing with cytoskeletal proteins (Zhang et al., 2015b). DPP9 can also modify the N-terminus of BRCA2 to promote repair of damaged DNA (Bolgi et al., 2022). Nevertheless, most studies observed association between altered DPP8/9 expression and prognosis of various types of tumors, but the specific underlaying mechanisms still need to be further explored.

The studies of DPP8/9 in the role of pyroptosis have made significant progress. The interaction of DPP9 with NLRP1 and CARD8 can inhibit their activation, thereby preventing the occurrence of pyroptosis (Huang et al., 2021a; Hollingsworth et al., 2021; Sharif et al., 2021). DPP8/9 inhibitors have been found to cause AML cell pyroptosis (Johnson et al., 2018), so DPP8/9 inhibitors may be potentially applicated in the treatment of hematological tumors. However, side effects of using DPP8/9 inhibitors such as pyroptosis of resting lymphocytes have been observed (Johnson et al., 2020; Linder et al., 2020). Therefore, the impact and safety of DPP8/9 inhibitors need to be further studied.

A variety of mechanisms are involved in the pathogenesis of kidney diseases, such as immune abnormalities, energy metabolism abnormalities, podocyte cytoskeleton structure damage. DPP8/9 has been implied involved in these mechanisms. The evidence of altered expression of DPP8/9 in the kidneys of CKD patients has suggested that DPP8/9 may play an essential role in the development and progression of kidney diseases (Zhang et al., 2021). However, only few data exploring the potential effects of DPP8/9 on kidney diseases are still available. Meantime, some studies have reported that global DPP9 enzyme inactivation is lethal in neonatal mice (Gall et al., 2013; Kim et al., 2017; Harapas et al., 2021) and experimental data on the effects of long-term DPP8/9 inhibitors application in animals are currently lacking. Therefore, studies on the potential pathophysiological effects of DPP8/9 and the application of their inhibitors in CKD will possibly shed light on the further understanding of the pathogenesis of CKD and explore the new potential therapeutical targets.

In conclusion, in this review we summarize the characteristics and functions of DPP8/9. The new research evidence of DPP8/9 have further supported that DPP8/9 are possible therapeutic targets for diseases such as tumors and inflammatory diseases. The advances have also revealed that DPP8/9 play a critical role in pyroptosis, organ fibrosis, COVID-19 infection, and so on. The regulation of DPP8/9 expression and enzymatic activity is expected to improve these pathogenic processes and diseases. The future studies based on the molecular structures of DPP8/9 and their roles in the physiological and pathophysiological processes will contribute to the further understanding of their properties, thereby facilitating the design of more specific small molecules or enzymatic inhibitors that compete with abnormal DPP8/9 expression or their enzymatic activity. Of note, the biological and pharmacological safety profiles of DPP8/9 inhibitors need further verification.
